# Intractable diarrhea caused by a rare rectal foreign body

**DOI:** 10.1002/kjm2.12917

**Published:** 2025-03-11

**Authors:** Qi Fan, Shuang Yu, De‐Hai Xiong

**Affiliations:** ^1^ Intestinal Center, Chongqing University Three Gorges Hospital Chongqing China; ^2^ Digestive Endoscopy Center, Chongqing University Three Gorges Hospital Chongqing China

Intrauterine device (IUD) is a safe, simple, effective, reversible, and economical contraceptive tool and is the most widely used method of contraception worldwide. However, some complications of IUD have been reported. One of these complications is transmural migration of the device to involve adjacent viscera such as peritoneum, vessels, bladder, small intestine, sigmoid colon, and rectum. Any displaced IUD should be removed due to potential complications.

A 44‐year‐old female presented with diarrhea repeatedly in the past year without abdominal pain and bloody stool. Oral antidiarrheal was ineffective. Rectal palpation was normal. Enteroscopy revealed a hard, immobile metallic rectal foreign body about 10 cm from the anus and the other colon mucosa was normal (Figure 1A). Abdominal CT showed a high density metal artifact at the right rear of the uterus, some of which entered the adjacent rectal cavity (Figure 1B). After asking about the past medical history, the patient had implanted an IUD more than 10 years ago. Subsequently, robot‐assisted laparoscopy for rectal foreign body removal (Figure 1C), rectal repair and uterine repair were performed and confirmed that the foreign object was a “V” IUD (Figure 1D). Postoperative anti‐infection and nutritional support were given and the patient recovered uneventfully. The patient completely rehabilitation with no complication observed during the 24‐month follow‐up.

**FIGURE 1:**
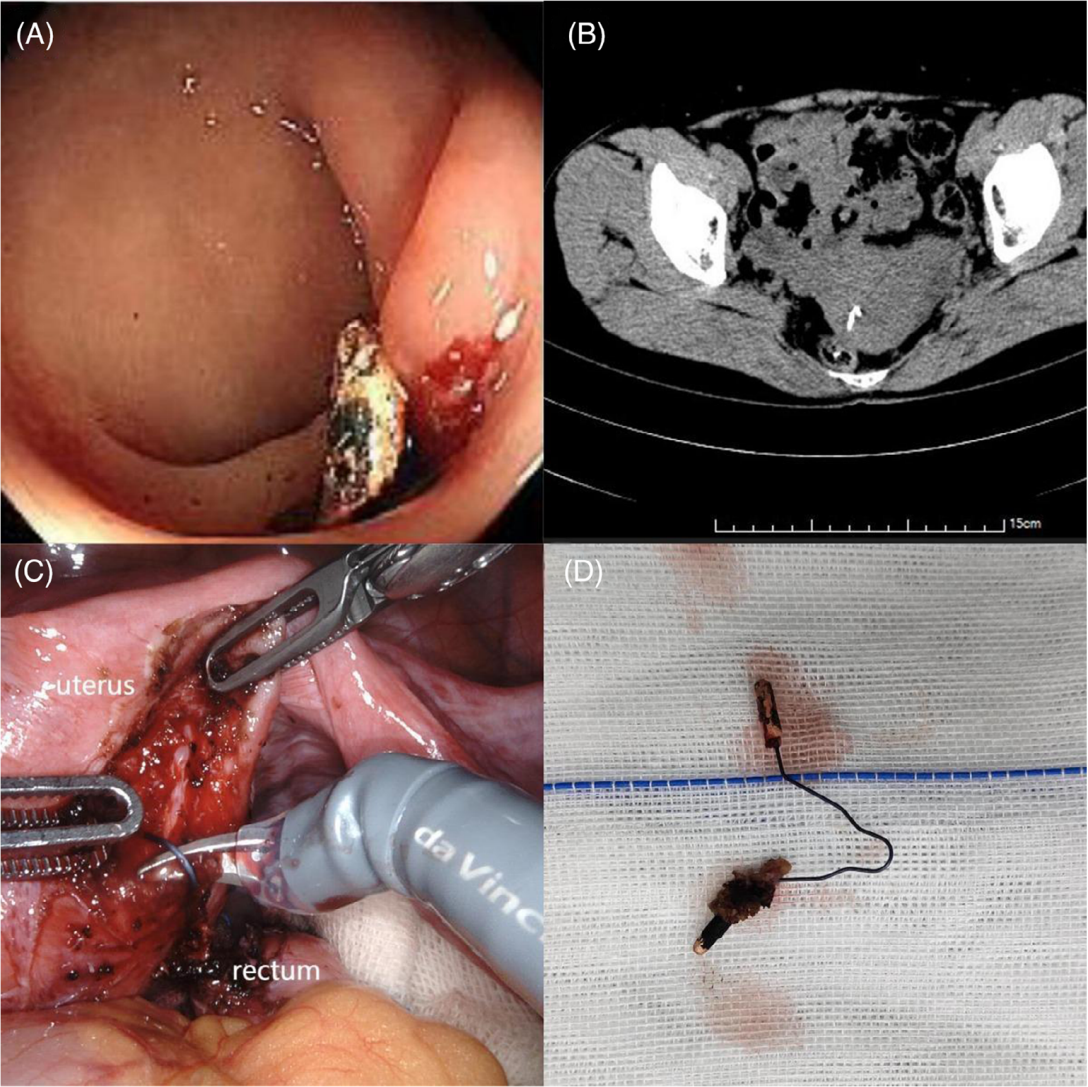
(A) Enteroscopy revealed a hard, immobile metallic rectal foreign body. (B) Abdominal CT showed a high density metal artifact at the right rear of the uterus, some of which entered the adjacent rectal cavity. (C) Robot‐assisted laparoscopy for rectal foreign body removal. (D) The foreign object was a “V” IUD.

IUD is a safe, simple, effective, reversible, and economical contraceptive tool and is the most widely used method of contraception worldwide. However, some complications of IUD have been reported. One of these complications is transmural migration of the device to involve adjacent viscera such as peritoneum, vessels, bladder, small intestine, sigmoid colon, and rectum.^1^, ^2^ Uterine Perforation and Rectal Migration is uncommon such as this case report. For female patients with a history of marriage and childbearing, they should be asked whether they have a history of IUD placement, and the complications caused by IUD displacement should be included in the differential diagnosis. Any displaced IUD should be removed due to potential complications.^3^, ^4^ WHO recommends surgical removal of the migrated IUD by minimally invasive methods, including hysteroscopy, cystoscopy, colonoscopy, or laparoscopy, depending on the location of the IUD.^5^

## CONFLICT OF INTEREST STATEMENT

The authors declare no conflicts of interest.
